# Hyperoside attenuates renal aging and injury induced by D-galactose via inhibiting AMPK-ULK1 signaling-mediated autophagy

**DOI:** 10.18632/aging.101723

**Published:** 2018-12-24

**Authors:** Buhui Liu, Yue Tu, Weiming He, Yinglu Liu, Wei Wu, Qijun Fang, Haitao Tang, Renmao Tang, Ziyue Wan, Wei Sun, Yigang Wan

**Affiliations:** 1 Department of Traditional Chinese Medicine, Nanjing Drum Tower Hospital Clinical College of Traditional Chinese and Western Medicine, Nanjing University of Chinese Medicine, Nanjing 210008, China; 2 Department of Nephrology, The Affiliated Hospital of Nanjing University of Chinese Medicine, Nanjing 210029, China; 3 Department of TCM Health Preservation, Second Clinic Medical School, Nanjing University of Chinese Medicine, Nanjing 210023, China; 4 Department of Traditional Chinese Medicine, Nanjing Drum Tower Hospital, The Affiliated Hospital of Nanjing University Medical School, Nanjing 210008, China; 5 Institute of Huangkui, Suzhong Pharmaceutical Group Co., Ltd., Taizhou 225500, China; 6 Department of Social Work, Meiji Gakuin University, Tokyo 108-8636, Japan; * Equal contribution

**Keywords:** hyperoside, renal aging, autophagy, AMPK-ULK1 signaling pathway, mTOR signaling pathway, vitamin E

## Abstract

The kidney is a typical organ undergoing age and injury. Hyperoside is reported to be useful for preventing aging induced by D-galactose (D-gal). However, therapeutic mechanisms remain unclear. We thereby aimed to verify whether hyperoside, compared to vitamin E (VE), could alleviate renal aging and injury by regulating autophagic activity and its related signaling pathways. *In vivo*, rats were administered with either hyperoside or VE after renal aging modeling induced by D-gal. Changes in renal aging and injury markers, autophagic activity and AMPK-ULK1 signaling pathway in the kidneys were analysed. *In vitro*, the NRK-52E cells exposed to D-gal were used to investigate regulative actions of hyperoside and VE on cell viability, renal tubular cellular aging markers, autophagic activity and its related signaling pathways by histomorphometry, immunohistochemistry, immunofluorescence, lentiviral transfection and Western blot. Aging and injury in the kidneys and renal tubular cells induced by D-gal were ameliorated by hyperoside and VE. Hyperoside and VE inhibited autophagic activity through mTOR-independent and AMPK-ULK1 signaling pathways. Hyperoside, as a component of phytomedicine similar to VE, attenuated renal aging and injury induced by D-gal via inhibiting AMPK-ULK1-mediated autophagy. This study provides the first evidence that hyperoside contributes to the prevention of age-associated renal injury.

## Introduction

Aging is defined as a decline in performance and fitness with advanced age [1,2]. The aging process is universal for almost all multicellular organisms, in which the kidney is a typical organ that undergoes age and injury characterized respectively by cell cycle arrest in G1 phase (p21/p53 proteins), shortened telomeres, staining for senescence-associated-β-galactosidase (SA-β-gal), inflammaging (the secretion of senescence-associated secretory phenotype, SASP) and renal interstitial fibrosis/tubular atrophy (IF/TA) [3–6]. Despite this, there is currently little *in vivo* and *in vitro* information on the therapeutic effects and mechanisms underlying renal age and injury. Xu et al. reported that klotho highly expressed in the kidneys and brain is an anti-aging gene encoding a single-pass transmembrane protein, which serves as an aging suppressor through a wide variety of mechanisms, including anti-oxidation, anti-senescence and modulation of many signaling pathways [7]. Many experiments confirmed that destruction of the klotho protein or loss of the klotho function leads to an accelerated aging. Therefore, the klotho protein may be a promising therapeutic target for renal age and injury [8]. Furthermore, recently, to analyze the pharmacological basis of anti-renal aging, several studies are developed. In these studies, calorie restriction (CR) has been shown to be the most robust nongenetic or pharmacological approach to understanding this phenomenon [9]. It has been reported that short-term CR may be considered a potential intervention for the retardation of renal aging by increasing autophagy and, subsequently, by reducing oxidative damage [10]. In addition, Calvo-Rubio et al. reported that long-term CR partially prevented or delayed the appearance of several structural hallmarks and autophagic processes in the aged kidneys [11]. Accordingly, Kume et al. also found that the reduced autophagy in the kidney might be involved in the age-associated weakness of proximal tubular cells (PTCs) against various renal lesions [12]. Undoubtedly, these results strongly suggest that autophagy, as the current focus of aging, should be helpful in the design of studies aiming to further explore anti-renal aging and should lead to the establishment of novel clinical treatments that may delay the progression of age-associated renal dysfunction in elderly patients.

Autophagy is a “self-eating” process to maintain intracellular homeostasis and cell integrity [13–16]. Autophagic dysfunction marked by the changes of light chain 3 I/II (LC3 I/II) and Beclin1 is involved in the pathogenesis of a variety of renal age and injury. The dynamic process of autophagy in all living cells, including PTCs, is usually surveyed by determining the autophagy flux [17,18]. Autophagy in renal age and injury is regulated by major nutrient-sensing pathways, including mammalian target of rapamycin (mTOR) complex 1, adenosine monophosphate-activated protein kinase (AMPK) and sirtuin 1 [19–21]. In addition, the class phosphatidylinositol-3-kinase (PI3K)/serine-threonine kinase (Akt) also activates the mTOR complex in response to insulin and other growth factors, acting as a negative regulator of autophagy. The activation of AMPK inhibits the mTORC1 complex and activates unc-51, similar to the autophagy activating kinase 1 (ULK1) complex. Thus, ULK1 and autophagy related gene (Atg) 13 phosphorylation act as positive regulators of autophagy in response to energy depletion [22–24]. More importantly, recently, Xu et al. found that D-galactose (D-gal) induces premature senescence in human lens epithelial cells (LECs) by impairing autophage flux and mitochondrial function *in vitro* [25]. Overall, targeting the activation of autophagy-related signaling axis triggered by D-gal, including the PI3K-Akt-mTOR and AMPK-ULK1 pathways in the kidneys, may reveal the therapeutic mechanisms for ameliorating renal age and injury *in vivo* and *in vitro*.

In China, traditional Chinese medicine (TCM) exerts anti-aging actions with unique dialectical treatment systems and multi-target mechanisms and has few adverse reactions [26]. Shen et al. reported that curcumin, an extract from turmeric, has vast potential in slowing down the process of aging and in combating age-related effects in model organisms [27]. Jafari et al. and Wiegant et al. reported that the extracts from *Rhodiola rosea* increased the longevity of worms and flies, respectively, without negative effects on reproduction or metabolic rate [28,29]. In addition, different from *turmeric* and *Rhodiola rosea*, the total flavonoid content from *abelmoschus manihot* (AM) (Huangkui capsule, the local name in China) and its component hyperoside (C_21_H_20_O_12_, CAS: 482-36-0) is reported to be useful for diminishing oxidative stress *in vivo* and for safely and effectively preventing premature senescence induced by D-gal [30]. However, until recently, there have still been some important issues that remain unresolved in terms of the role of renal age and injury treated by hyperoside, a component of AM. For instance, questions of whether hyperoside improves renal aging and injury by means of targeting autophagic signaling, such as the PI3K-Akt-mTOR and/or AMPK-ULK1 pathways, as well as the underlying therapeutic mechanisms involved *in vivo* and *in vitro*, remain.

Thus, here, to address these issues, we designed animal and cell experiments to test these hypotheses that hyperoside alleviates D-gal-induced renal aging and injury by regulating autophagic activity and its related signaling pathways *in vivo* and *in vitro*, and the results were compared to the classic anti-aging drug vitamin E (VE) [31–34]. The results were in agreement with the hypothesis and suggested that the regulation of autophagy was protective in age-associated renal injury.

## RESULTS

### Renal aging and injury induced by D-galactose are improved by hyperoside and vitamin E *in vivo*

**Figure 1:**
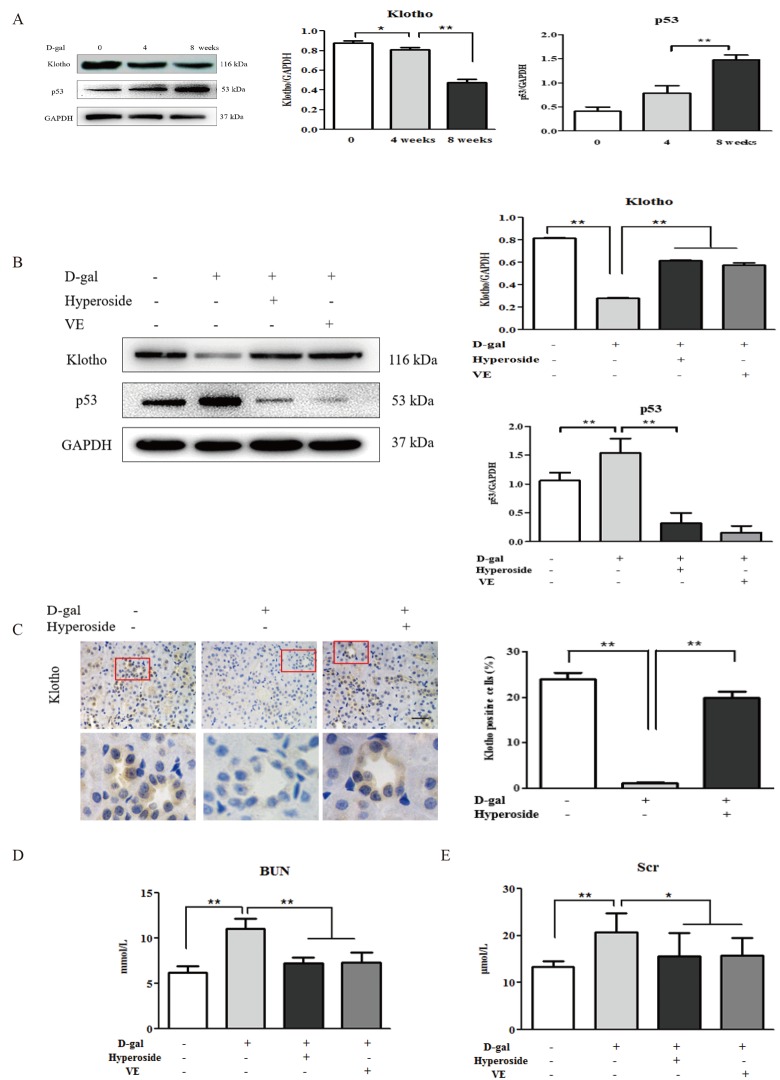
**The effects of hyperoside and vitamin E on renal aging and injury induced by D-galactose *in vivo*.** (**A**) A WB analysis of klotho and p53 in the kidneys of the rats in the control, the 4 week-D-gal and the 8 week-D-gal groups. (**B**) A WB analysis of klotho and p53 in the kidneys of the rats in the control, the 8 week-D-gal, the D-gal + Hyperoside and the D-gal + VE groups. (**C**) Immunohistochemical staining of klotho and the percentage of positively stained areas for klotho in the control, the 8 week-D-gal and the D-gal + Hyperoside groups. Scale bar = 20 μm. (**D**, **E**) The serum levels of BUN and Scr in the control, the 8 week-D-gal, the D-gal + Hyperoside and the D-gal + VE groups. The data are expressed as the mean ± SD, (n=3), ^*^P < 0.05, ^**^P < 0.01. Abbreviation: WB, Western blot; D-gal, D-galactose; VE, vitamin E; BUN, blood urea nitrogen; Scr, serum creatinine.

First, we examined the dynamic changes in the protein expression of klotho as a representative aging suppressor [8] and p53 (a cell cycle arrest protein) as a known hallmark of aging [35] in the kidneys of the D-gal-treated model rats. Compared to the control group, the protein expression levels of klotho and p53 in the kidneys were mildly changed in the 4 week-D-gal group. After the D-gal treatment for 8 weeks (the 8 week-D-gal group), the protein expression level of klotho in the kidneys was significantly downregulated, whereas, the protein expression level of p53 in the kidneys was obviously upregulated, compared to the 4 week-D-gal group, respectively (Figure 1A). Then, we confirmed whether hyperoside and VE ameliorated renal aging and injury in the D-gal-treated model rats. In Figure 1B, the changed protein expression levels of klotho and p53 in the kidneys of the D-gal-treated model rats were notably ameliorated in both the D-gal + Hyperoside and the D-gal + VE groups compared to the 8 week-D-gal group. Moreover, the number of positive cells in the klotho immunohistochemical staining in the renal tubular areas of the D-gal-treated model rats was significantly increased after the treatment of hyperoside for 8 weeks compared to the 8 week-D-gal group (Figure 1C). In addition, the slightly increased levels of blood urea nitrogen (BUN) and serum creatinine (Scr) in the D-gal-treated model rats were simultaneously detected, and the decreased levels of BUN and Scr were found after the treatment with hyperoside and VE for 8 weeks compared to the 8 week-D-gal group (Figure 1D, E).

### Renal cellular aging and injury are induced by D-galactose *in vitro*

**Figure 2:**
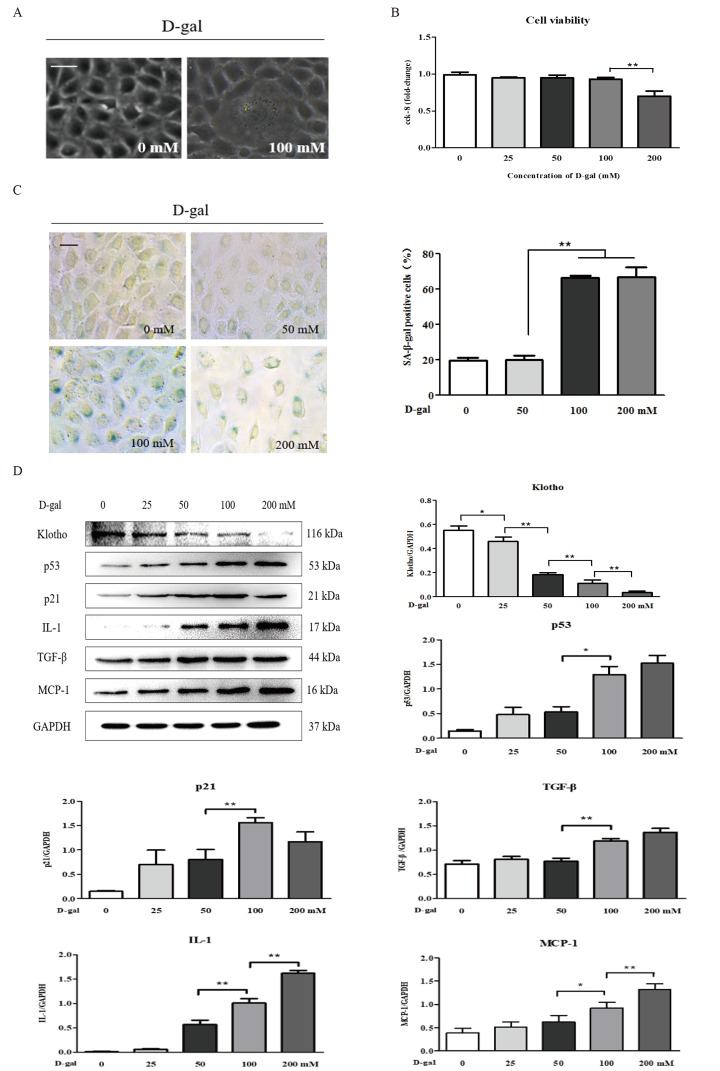
**The characteristics of renal cellular aging and injury** **induced by D-galactose *in vitro*.** (**A**) The morphological changes in the NRK-52E cells with or without 100 mM D-gal treatment for 24 hours by light microscope. (**B**) The cell viability in the NRK-52E cells exposed to D-gal at 0, 25, 50, 100, and 200 mM for 24 hours. (**C**) SA-β-gal staining in the NRK-52E cells exposed to D-gal at 0, 50, 100, and 200 mM for 24 hours, and the percentage of SA-β-gal-positive cells. (**D**) The NRK-52E cells were treated with D-gal at 0, 25, 50, 100, and 200 mM for 24 hours, and subjected to a WB analysis of klotho, p53, p21, IL-1, TGF-β and MCP-1. The data are expressed as the mean ± SD, (n=3), ^*^P < 0.05, ^**^P < 0.01. Abbreviation: D-gal, D-galactose; SA-β-gal, senescence-associated-β-galactosidase; WB, Western blot; IL-1, interleukin-1; TGF-β, transforming growth factor-β; MCP-1, monocyte chemoattractant protein-1.

D-gal induced renal aging and injury *in vivo*. Next, we detected whether D-gal induced renal tubular cellular aging and injury *in vitro*. Using a light microscope, we found that D-gal, at a dose of 100 mM, induced cellular aging and injury in the NRK-52E cells, as characterized by the enlarged volume and the vacuolation of cell nucleus (Figure 2A), but it did not affect the cell viability (Figure 2B). In Figure 2C, the strong positive staining for SA-β-gal was observed in the NRK-52E cells treated with D-gal at the doses of 100 mM and 200 mM, along with a significant increase in the percentage of SA-β-gal-positive cells compared to the control group. Here, it was noted that the 200 mM dose of D-gal reduced the cell viability significantly compared to the 100 mM dose of D-gal (Figure 2B). In addition, with the treatment of D-gal at the different doses in the NRK-52E cells, the protein expression level of klotho was downregulated significantly, in a dose-dependent manner, on the contrary, the protein expression levels of p21, p53, interleukin (IL)-1, transforming growth factor (TGF)-β and monocyte chemoattractant protein (MCP)-1 (the hallmarks of inflammaging) were obviously upregulated respectively, also in a dose-dependent manner (Figure 2D).

### Renal cellular aging and injury are ameliorated by hyperoside and vitamin E *in vitro*

**Figure 3:**
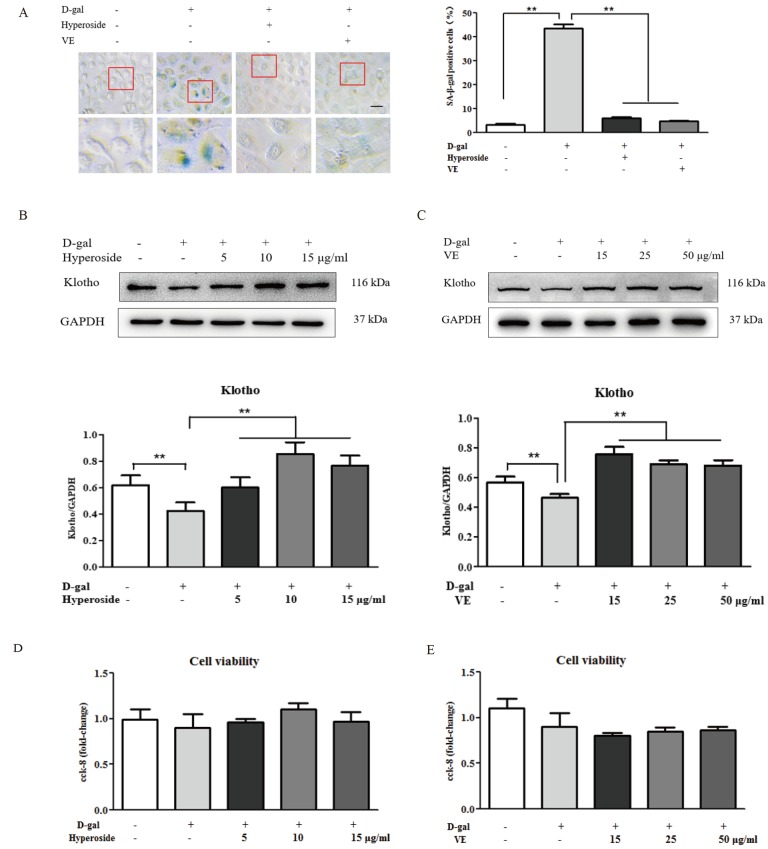
**The effects of hyperoside and vitamin E on renal cellular aging and injury *in vitro*.** (**A**) SA-β-gal staining in the NRK-52E cells exposed to D-gal at 100 mM, with the treatment of hyperoside at 10 μg/ml or VE at 25 μg/ml for 24 hours, and the percentage of SA-β-gal-positive cells. (**B**, **C**) The NRK-52E cells were exposed to D-gal at 100 mM, with the treatment of hyperoside at 0, 5, 10, and 15 μg/ml and VE at 0, 15, 25, and 50 μg/ml for 24 hours, and subjected to a WB analysis for klotho, respectively. (**D**, **E**) The cell viability in the NRK-52E cells exposed to D-gal at 100 mM, with the treatment of hyperoside at 0, 5, 10, and 15 μg/ml and VE at 0, 15, 25, and 50 μg/ml for 24 hours. The data are expressed as the mean ± SD, (n=3), ^**^P < 0.01. Abbreviation: SA-β-gal, senescence-associated-β-galactosidase; D-gal, D-galactose; VE, vitamin E, WB, Western blot.

**Figure 4:**
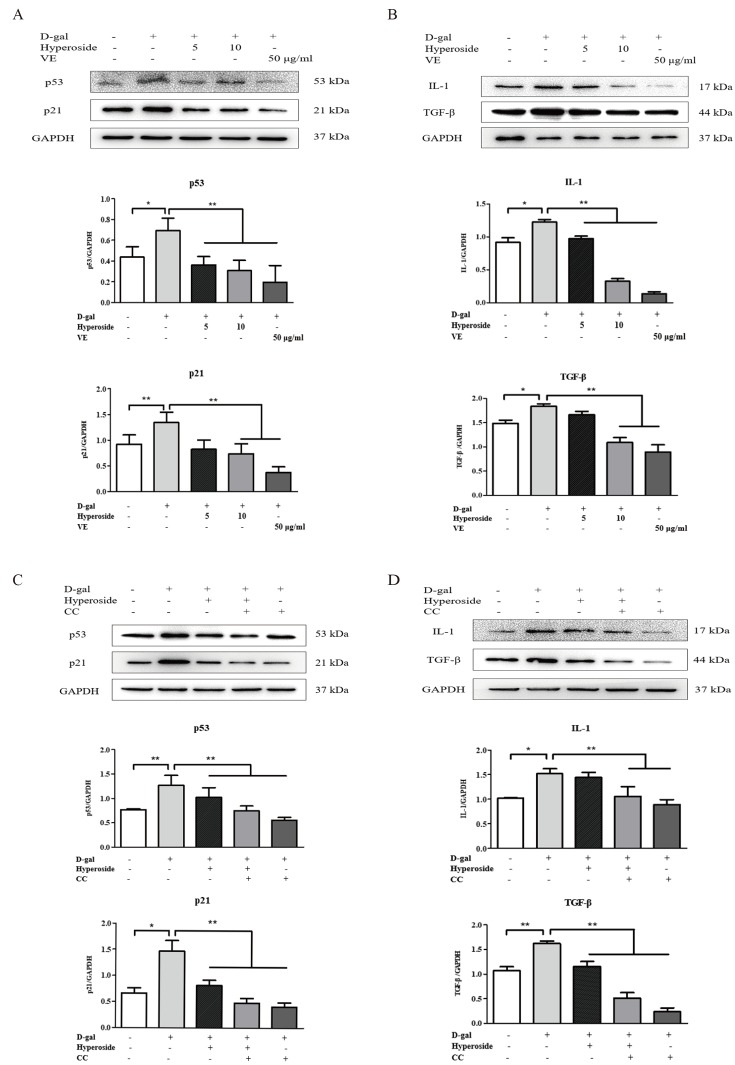
**The actions of hyperoside, vitamin E and compound C on renal cellular aging** **and injury *in vitro*.** (**A**, **B**) The NRK-52E cells were exposed to D-gal at 100 mM, with the treatment of hyperoside at 0, 5, and 10 μg/ml and VE at 50 μg/ml for 24 hours, and subjected to a WB analysis for p53, p21, IL-1 and TGF-β, respectively. (**C**, **D**) The NRK-52E cells were exposed to D-gal, with the treatment of hyperoside and CC for 24 hours, and subjected to a WB analysis for p53, p21, IL-1 and TGF-β, respectively. The data are expressed as the mean ± SD, (n=3), ^*^P < 0.05, ^**^P < 0.01. Abbreviation: D-gal, D-galactose; VE, vitamin E; WB, Western blot; IL-1, interleukin-1; TGF-β, transforming growth factor-β; CC, compound C.

Hyperoside and VE alleviated D-gal-induced renal aging and injury *in vivo*, and we further investigated whether hyperoside and VE attenuated renal tubular cellular aging and injury induced by D-gal *in vitro*. In Figure 3A-C and Figure 4A, B, both the strong SA-β-gal positive staining and the changed protein expression levels of klotho, p21, p53, IL-1 and TGF-β induced by D-gal in the NRK-52E cells were significantly improved after the co-treatment of D-gal and hyperoside or VE compared to the control group. However, the co-treatment of D-gal and hyperoside or VE at the different doses did not significantly affect the cell viability in the NRK-52E cells (Figure 3D, E). In addition, the co-treatment of D-gal and compound C (CC, a pharmacologic inhibitor of AMPK), similar to hyperoside or VE, also decreased the protein expression levels of p21, p53, IL-1 and TGF-β induced by D-gal in the NRK-52E cells compared to the control group (Figure 4C, D).

### Autophagic activity is inhibited by hyperoside and vitamin E through the mTOR-independent and AMPK-ULK1 signaling pathways *in vitro*

**Figure 5:**
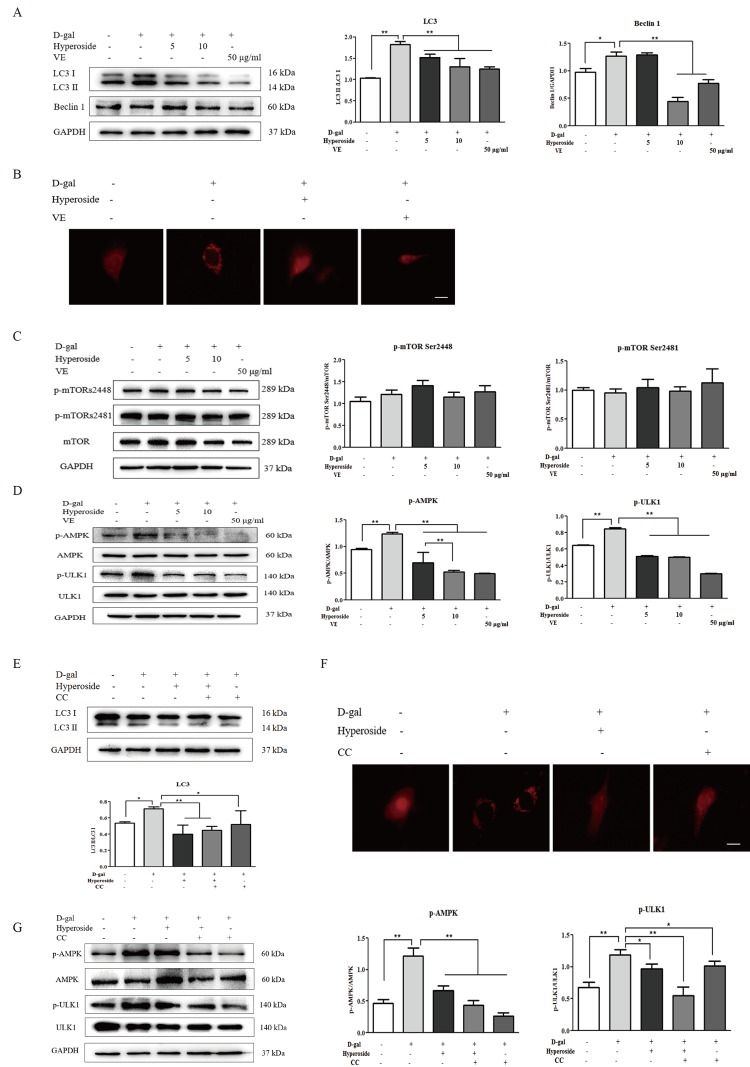
**The effects of hyperoside and vitamin E on autophagic activity and the mTOR-independent and AMPK-ULK1 signaling pathways *in vitro*.** (**A**) The NRK-52E cells were exposed to D-gal at 100 mM, with or without the treatment of hyperoside at 5 and 10 μg/ml or VE at 50 μg/ml for 24 hours, and subjected to a WB analysis for LC3 I/II and Beclin1. (**B**) The NRK-52E cells were infected with the RFP-LC3 Lentiviral Biosensor, exposed to D-gal at 100 mM with or without the treatment of hyperoside at 10 μg/ml or VE at 50 μg/ml for 24 hours and subjected to fluorescence microscopy. Scale bar = 5 μm. (**C, D**) The NRK-52E cells were exposed to D-gal at 100 mM with or without the treatment of hyperoside at 5 and 10 μg/ml or VE at 50 μg/ml for 24 hours, and subjected to a WB analysis for p-mTOR (Ser2448), p-mTOR (Ser2481) and mTOR (C1), as well as p-AMPK, AMPK, p-ULK1 and ULK1. (**E**) The NRK-52E cells were exposed to D-gal at 100 mM with or without the treatment of hyperoside at 10 μg/ml and CC at 10 μM for 24 hours, and subjected to a WB analysis for LC3 I/II. (**F**) The NRK-52E cells were infected with the RFP-LC3 Lentiviral Biosensor, exposed to D-gal at 100 mM with or without the treatment of hyperoside at 10 μg/ml and CC at 10 μM for 24 hours and subjected to fluorescence microscopy. Scale bar = 5 μm. (**G**) The NRK-52E cells were exposed to D-gal at 100 mM with or without the treatment of hyperoside at 10 μg/ml and CC at 10 μM for 24 hours, and subjected to a WB analysis for p-AMPK, AMPK, p-ULK1 and ULK1. The data are expressed as the mean ± SD, (n=3), ^*^P < 0.05, ^**^P < 0.01. Abbreviation: D-gal, D-galactose; VE, vitamin E; WB, Western blot; p-mTOR, phosphorylated mTOR; p-AMPK, phosphorylated AMPK; p-ULK1, phosphorylated ULK1; CC, compound C.

Recent studies show that autophagy plays a crucial role in the regulation of renal aging [15,16]. Therefore, we first examined the protein expression levels of LC3 I/II (autophagic active marker) and Beclin1 in the NRK-52E cells treated with D-gal alone or with the co-treatment of D-gal and hyperoside or VE. In Figure 5A, D-gal significantly induced the conversion of LC3 (LC3 I to LC3 II) and the protein expression level of Beclin1. With the treatment of hyperoside at the different doses or VE in the NRK-52E cells exposed to D-gal, both the conversion of LC3 and the protein expression level of Beclin1 were significantly decreased compared to the control group. Moreover, it is noteworthy that D-gal induced a number of punctate dots in the NRK-52E cells infected with the RFP-LC3 Lentiviral Biosensor, while these D-gal-induced LC3-labelled punctate dots were noticeably decreased after the co-treatment of D-gal and hyperoside or VE (Figure 5B).

mTOR signaling is an important regulatory mechanism in autophagy [17]. Thus, we next tested the protein expression levels of the key molecules involved in mTOR signaling, including p-mTOR Ser2448, p-mTOR Ser2481 and mTOR, in the NRK-52E cells. In Figure 5C, there was no significant change in the protein expression levels of p-mTOR Ser2448, p-mTOR Ser2481 and mTOR in the NRK-52E cells exposed to D-gal alone or with the co-treatment of D-gal and hyperoside or VE.

AMPK is a serine/threonine protein kinase that acts as a positive regulator of autophagy [18]. ULK1, as the downstream substrate of AMPK, stimulates the engulfment of damaged proteins and organelles in autophagosomes and activates autophagic process [20–22]. Hence, we next observed the changes in AMPK-ULK1 signaling. In Figure 5D, D-gal markedly increased the phosphorylation of AMPK and ULK1 (p-AMPK and p-ULK1) in the NRK-52E cells, which was significantly decreased by the co-treatment of D-gal and hyperoside, in a dose-dependent manner, or VE compared to the control group. Further, we used CC (AMPK inhibitor) to examine the role of AMPK-ULK1 signaling in the NRK-52E cells. In Figure 5E and F, the co-treatment of hyperoside and CC significantly reduced the conversion of LC3 induced by D-gal and markedly inhibited the LC3-labelled punctate dots induced by D-gal. Furthermore, the protein expression levels of p-AMPK and p-ULK1 were significantly downregulated after the co-treatment of hyperoside and CC in the NRK-52E cells exposed to D-gal compared to the control group (Figure 5G).

### Autophagic activity is similarly depressed by hyperoside and vitamin E through the AMPK-ULK1 signaling pathway *in vivo*

**Figure 6:**
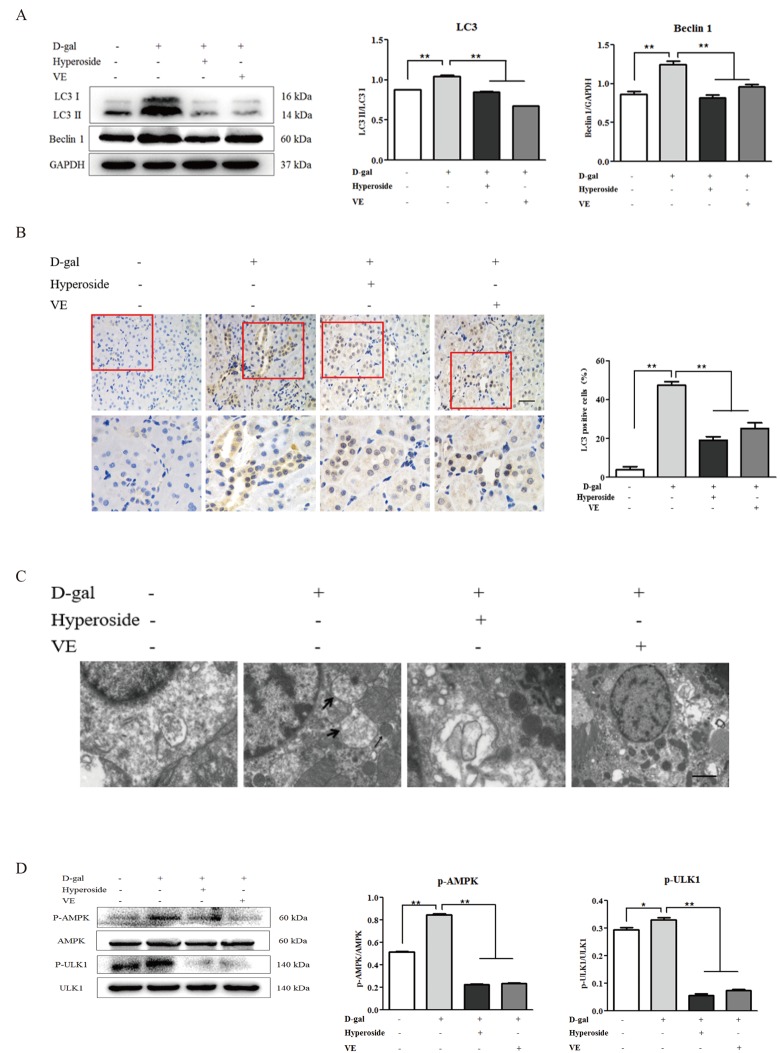
**The effects of hyperoside and vitamin E on autophagic activity and the AMPK-ULK1 signaling pathway *in vivo*.** (**A**) A WB analysis of LC3 I/II and Beclin1 in the kidneys from the rats in the control, the 8 week-D-gal, the D-gal + Hyperoside and the D-gal + VE groups. (**B**) Immunohistochemical staining of LC3 and the percentage of the positively stained areas of LC3 in the control, the 8 week-D-gal, and the D-gal + Hyperoside groups. Scale bar = 20 μm. (**C**) The morphological changes in the renal tubular cells of the rats in the control, the 8 weeks-D-gal, the D-gal + Hyperoside and the D-gal + VE groups by transmission electron microscopy. The black arrows show the autophagosomes with the characteristic morphology of a double membrane. (**D**) A WB analysis of p-AMPK, AMPK, p-ULK1 and ULK1 in the kidneys of the rats in the control, the 8 weeks-D-gal, the D-gal + Hyperoside and the D-gal + VE groups. The data are expressed as the mean ± SD, (n=3), ^*^P < 0.05, ^**^P < 0.01. Abbreviation: WB, Western blot; D-gal, D-galactose; VE, vitamin E; p-AMPK, phosphorylated AMPK; p-ULK1, phosphorylated ULK1.

Since hyperoside and VE suppressed D-gal-induced autophagic activity by inhibiting AMPK-ULK1 signaling *in vitro*, we suspected that hyperoside and VE might also ameliorate D-gal-induced renal aging via regulating autophagic activity as well as AMPK-ULK1 signaling in the kidneys of the D-gal-treated model rats. In Figure 6A, we found that the conversion of LC3 and the protein expression level of Beclin1 in the kidneys of the D-gal-induced renal aging rats were obviously increased, and decreased significantly after the treatment of hyperoside and VE compared to the 8 week-D-gal group. Additionally, the similar changes were also found in the number of positive cells after LC3 immunohistochemical staining in the renal tubular areas (Figure 6B). Here, using a transmission electron microscope, typical autophagosomes, with the characteristic morphology of a double membrane, were observed in the D-gal-induced renal aging rats compared to the control group, while hyperoside and VE decreased the number of autophagosomes compared to the 8 week-D-gal group (Figure 6C). In addition to these results, compared to the 8 week-D-gal group, hyperoside and VE significantly inhibited the phosphorylation of AMPK and ULK1 by downregulating the protein expression levels of p-AMPK and p-ULK1 in the kidneys of the D-gal-induced renal aging rats (Figure 6D).

## DISCUSSION

In the present study, we demonstrated the following: D-gal induced aging and injury in the kidneys and renal tubular cells, which were ameliorated by the treatment of hyperoside and VE *in vivo* and *in vitro*; Hyperoside and VE inhibited autophagic activity induced by D-gal through the mTOR-independent and AMPK-ULK1 signaling pathways *in vitro*; Hyperoside and VE also depressed autophagic activity induced by D-gal through the AMPK-ULK1 signaling *in vivo*. Thus, hyperoside and VE alleviated renal aging and injury *in vivo* and *in vitro* by inhibiting autophagic activity and AMPK-ULK1 signaling.

To explain the therapeutic effects and mechanisms of anti-aging drugs on visceral organ aging, the several experimental animal models of aging are widely used. Most *in vivo* research on renal aging has been performed in rats and mice. Generally, wild-type mice reveal milder age-associated renal damage, whereas all conventional strains of rats develop more obvious age-associated renal injury, with proteinuria and renal dysfunction. Correspondingly, Lei et al. and Park et al. reported that free radical production was significantly increased in the kidney and other tissues when rats were injected with D-gal for 6 weeks [36,37]. Therefore, D-gal has been used to induce oxidative injuries *in vivo* to mimic the natural aging process in rats. In this study, we first established the renal aging rat model by a subcutaneous injection of D-gal at a high dose. Our results showed that, compared to the control group rats, after modeling for 8 weeks, the changes of aging and injury-associated hallmarks in the kidney, the typical renal tubular damage and the slight decline of renal function in the D-gal-treated model rats were detected concurrently. In addition, *in vitro*, the features of renal tubular cellular aging and injury were also found in the NRK-52E cells treated with D-gal. More importantly, these characteristic injurious changes in renal aging and renal tubular cellular aging were both ameliorated by the treatment of hyperoside and VE *in vivo* and *in vitro*. Therefore, we considered that the animal and cellular aging models induced by D-gal should be helpful in unravelling the mechanisms of renal aging and in finding novel therapeutic drugs for renal aging and injury in elderly patients.

To our knowledge, D-gal, as a reducing monosaccharide abundant in milk products and other non-dairy food, is widely used in the various age-related disorders, which are partially elucidated to be associated with autophagic dysfunction [25,38,39]. Hence, targeting autophagic activity, we then investigated the changes in autophagic markers, including LC3 I/II and Beclin1, in the NRK-52E cells treated with D-gal alone and co-treated with D-gal and hyperoside or VE, as well as in the kidneys of the D-gal-treated model rats. The results in this study revealed that, compared to the normal NRK-52E cells *in vitro*, D-gal not only induced renal tubular cellular aging but also triggered autophagic activity, as characterized by LC3 conversion, Beclin1 protein expression and RFP-LC3-labelled punctate dots in the NRK-52E cells. *In vivo*, compared to the control group, D-gal also improved renal aging and induced autophage-related changes in the kidneys of the D-gal-treated model rats, including LC3 conversion, Beclin1 protein expression, LC3 immunohistochemical staining and autophagosomes formation. In addition to these results, more importantly, we confirmed that hyperoside and VE inhibited autophagic activity induced by D-gal *in vivo* and *in vitro*.

Autophagy, a protein degradation system, is present at a basal level in all mammals and is regulated by the nutrient-sensing-related signaling pathways, such as mTOR, AMPK and sirtuin 1 [17]. mTOR is the key regulator of cell growth, metabolism, survival and the lifespan of organisms; therefore, it is closely and negatively associated with autophagy [19]. Our previous study demonstrated that the phosphorylation of mTOR Ser2448 decreased in the NRK-52E cells following starvation-induced by Hank’s balanced salt solution in a time-dependent manner [40]. Recently, Yuan et al. also reported that autophagy was regulated through mTOR suppression in the degeneration of the auditory cortex [41]. Hence, we then examined the protein expression levels of the key molecules in mTOR signaling, including p-mTOR Ser2448, p-mTOR Ser2481 and mTOR, as well as its downstream regulators, including 70-kDa ribosomal protein S6 (p70S6K) and eukaryotic initiation factor 4E-binding protein-1 (4EBP1), in the NRK-52E cells. Unexpectedly, no significant changes in mTOR signaling and its downstream regulators, p70S6K and 4EBP1 (data not shown), were shown in the NRK-52E cells exposed to D-gal alone or with the co-treatment of D-gal and hyperoside or VE. Thus, we naturally assumed that autophagic activity induced by D-gal might be regulated by hyperoside and VE through mTOR-independent signaling pathways. Unlike mTOR, AMPK, as a positive regulator of autophagy, is activated and stimulates autophagy by directly activating ULK1, which initiates the process of autophagy [23]. Wang et al. reported that triclosan induces autophagy in non-phagocytic cells and in macrophages via the AMPK/ULK1 pathway, independent of mTOR [42]. Accordingly, we further hypothesized that autophagic activity might be inhibited by hyperoside and VE through the AMPK/ULK1 signaling pathway. Our results indicated that D-gal induced the phosphorylation of AMPK and ULK1 in the aged NRK-52E cells and in the kidneys of the renal aging model rats. In addition, the increased protein expression levels of p-AMPK and p-ULK1 were significantly decreased by the treatment of hyperoside or VE both *in vitro* and *in vivo*. It should be stressed here that, to further validate that hyperoside and VE could inhibit autophagic activity through the AMPK/ULK1 signaling pathway, CC, a well-known inhibitor of AMPK, was used in the *in vitro* experiments. We found that the co-treatment of hyperoside and CC not only reduced autophagic activity by decreasing LC3 conversion but also downregulated the protein expression levels of p-AMPK and p-ULK1 in the aged NRK-52E cells exposed to D-gal. More importantly, we also detected that AMPK inhibitor CC ameliorated the aged NRK-52E cells induced by D-gal. Collectively, these results strongly suggested that hyperoside and VE inhibited autophagic activity induced by D-gal through the AMPK-ULK1 signaling pathway *in vitro* and *in vivo*, without affecting the classic mTOR signaling pathway.

Finally, we need to discuss 2 additional points. First, why could not the increased autophagic activity induced by D-gal protect against renal aging? Indeed, the positive crosstalk between aging and autophagy has been extensively reported. However, thus far, the functional relationship between aging and autophagic activity is still inconclusive. It has been reported that mice with a specific autophagy deficiency in renal proximal tubular cells have an increased accumulation of damaged mitochondria and ubiquitinated proteins associated with tubular apoptosis and fibrosis, leading to premature senescence [43,44]. However, Xu et al. proved that autophagic flux was limited in the aging process induced by D-gal due to a declined efficiency in autophagic degradation in human LECs [25]. Our finding also suggested that autophagic activity triggered senescence in the aged NRK-52E cells and in the kidneys of the renal aging model rats treated by D-gal *in vitro* and *in vivo*. Autophagy is an important defence response against aging in the early stage of senescence, whereas it is also positively related to aging in the middle and advanced stages of senescence [45,46]. Second, why were the anti-aging effects *in vivo* and *in vitro* of hyperoside similar to VE? It is well-known that aging can be induced by inflammation and oxidative stress, which cause cellular damage and senescence. VE (α-tocopherol) reacts rapidly with organic free radicals and is widely accepted as having antioxidant properties [47]. In addition, VE not only exhibits potent anti-proliferative effects on human tumour cells but also triggers an autophagic process in human gastric cancer SGC-7901 cells via Akt/AMPK/mTOR signaling [48]. Therefore, it is believed that VE, as an antioxidant, protects against aging by intervening in oxidative stress and autophagy. In a similar way, hyperoside, as a component of AM, is reported to attenuate renal damage in cisplatin-induced acute kidney injury by inhibiting inflammation and oxidative stress [49]. Furthermore, the results in our study also suggested that hyperoside and VE alleviated renal aging and renal tubular cellular aging by the same AMPK-ULK1 signaling-mediated autophagy pathway. Further studies of the pharmacological similarities between hyperoside and VE, with regard to anti-aging, are needed in the future.

In summary, we demonstrated that hyperoside, as a component of phytomedicine similar to VE, attenuated renal aging and injury induced by D-gal via inhibiting AMPK-ULK1 signaling-mediated autophagy. This study provides the first evidence that hyperoside contributes to the prevention of age-associated renal injury.

## MATERIALS AND METHODS

### Reagents

D-gal was obtained from Sigma-Aldrich Chemical Co. (St Louis, MO, USA). Hyperoside was obtained from Liangwei Bioscience Co., Ltd. (Nanjing, China). VE used in the *in vivo* experiments was obtained from Yuanye Bioscience Co., Ltd. (Shanghai, China), while VE used in the *in vitro* experiments was obtained from Maibo Bioscience Co., Ltd. (Nanjing, China). Compound C, an inhibitor of AMPK, was obtained from Selleckchem (Houston, TX, USA).

### Animal experiments

Twenty-six Sprague-Dawley (SD) male rats, weighing approximately 200 g each, were purchased from the Animal Center of Nanjing Medical University (Nanjing, China). The experiments were performed in accordance with protocols approved by the Animal Ethics Committee of Nanjing University Medical School [Permit Number: SCXK (SU) 2016-0002]. All the rats were housed with conditions of 22 ± 3 °C and 50 ± 10% humidity using a 12-hour light/dark cycle, and they were fed a standard rat chow and given tap water *ad libitum* in the Experimental Animal Center of The Affiliated Hospital of Nanjing University Medical School (Nanjing Drum Tower Hospital). The rats were given 1 week to acclimatize before the experiment.

The rats were divided into 5 groups, according to a random number table, as follows: 5 rats in the control group (distilled water for 8 weeks), 3 rats in the 4 week-D-gal group (D-gal for 4 weeks), 6 rats in the 8 week-D-gal group (D-gal for 8 weeks), 6 rats in the D-gal + Hyperoside group (D-gal + Hyperoside for 8 weeks), and 6 rats in the D-gal + VE group (D-gal + VE for 8 weeks). The rats were given D-gal at a dosage of 300 mg/kg/d by a subcutaneous injection, while hyperoside, at a dosage of 20 mg/kg/d, and VE, at a dosage of 30 mg/kg/d, were given by oral administration. At the end of 4 weeks, the rats in the 4 week-D-gal group were killed, while at the end of 8 weeks, all the other rats were killed. All the rats were anaesthetized by an intraperitoneal injection of ketamine and diazepam (1:1) and were sacrificed by cardiac puncture. The blood serum and kidneys were collected for the detection of various indicators.

### Renal function analysis

BUN and Scr were measured using an automatic biochemical analyser in the Department of Laboratory Medicine of Nanjing Drum Tower Hospital.

### Western blot analysis

A Western blot (WB) analysis was performed as described previously [50]. The level of klotho was assessed using an anti-klotho antibody (Abcam, Cambridge, MA). The levels of p21, p53, IL-1, TGF-β and MCP-1 were assessed using anti-p21, p53, IL-1, TGF-β and MCP-1 antibodies, respectively (Abcam, Cambridge, MA). The levels of LC3 I/II and Beclin1 were assessed using anti-LC3A/B and anti-Beclin1 antibodies, respectively (Cell Signaling, Beverly, MA). The levels of the phosphorylated and total proteins for mTOR were assessed using anti-phospho mTOR Ser2448, anti-phospho mTOR Ser2481 and anti-mTOR antibodies (Cell Signaling, Beverly, MA, USA). The levels of the phosphorylated and total proteins of AMPK and ULK1 were assessed using anti-phospho AMPKα Thr172, anti-AMPKα, anti-phospho ULK1 and anti-ULK1 antibodies (Cell Signaling, Beverly, MA, USA). The level of glyceraldehyde-3-phosphate dehydrogenase (GAPDH) was assessed using an anti-GAPDH antibody (Bioworld Technology Inc., MN, USA) as a loading control. The blots were visualized using an enhanced chemiluminescence detection system (Tanon-5200Muilti, China). The densitometric analysis was performed using ImageJ Software.

### Immunohistochemistry assay

Immunohistochemistry was performed as described previously [40]. The renal tissue slides were incubated with primary antibodies against klotho (Abcam, Cambridge, MA) and LC3 I/II (Cell Signaling, Beverly, MA) and secondary horseradish peroxidase (HRP)-conjugated anti-rabbit immunoglobulins (Abcam, New Territories, HK). Using light microscopy, changes in the kidneys and the positively stained areas were observed. These positive areas were visualized at a magnification of 200 ×, and the percentages of the positive areas in the whole renal areas were calculated in 3 randomly selected, nonoverlapping fields with Image-Pro Plus 5.0 software (Media Cybernetics, Silver Spring, MD).

### Cell culture

The NRK-52E cells, a rat renal proximal tubular epithelial cell line, were cultured in Dulbecco’s modified Eagle’s medium/Ham’s F-12 (HyClone) supplemented with 5% foetal bovine serum (FBS; Gibco, Grand Island, NY).

### Light microscope examination

The NRK-52E cells were treated with or without 100 mM D-gal for 24 hours. The morphological changes were captured at 200 × magnification, using a Leica-DMIL light microscope (Germany).

### Cell viability assessment

The cell viability was assessed using CCK-8 (Beyotime, Shanghai, China). The cells were seeded into 96-well plates, with 3 replicate wells for each group, at a density of 1×10^4^ cells per well, with 100 μl medium. After the cells were incubated for the indicated time, 10 μl of the CCK-8 solution was added to each well, followed by an incubation for 2 hours. The optical density (OD) was computed at the absorbance of 450 nm, and the cell viability was calculated.

### Senescence-associated-β-galactosidase staining assay

Intracellular SA-β-gal staining was performed with the SA-β-gal Staining Kit (Beyotime, Shanghai, China) according to the manufacturer’s instructions. The aging cells were identified by a bluish green-stain under a light microscope. The percentage of positive-stained cells in the total cells was counted in 3 random fields under a microscope at 200 × magnification.

### Lentiviral transfection

The NRK-52E cells were seeded onto 12-well glass chamber slides in growth medium and were cultured until they were approximately 60% confluent. The medium was replaced with the fresh growth medium the next day, and the RFP-LC3 Lentiviral Biosensor (LentiBrite™, Millipore, USA) was added at 50 multiplicity of infection (MOI). The MOI refers to the ratio of the number of infectious lentiviral particles to the number of cells being infected. The infected NRK-52E cells were then incubated for 24 hours. After the lentiviral transfection, the lentivirus-containing medium was removed and was replaced with the fresh growth medium. The infected cells were cultured for another 48 hours, and the medium was changed every 24 hours.

### Fluorescence microscopy assay

The infected NRK-52E cells were exposed to D-gal with or without hyperoside, VE or CC for 24 hours. The confocal images were captured at 200 × magnification, using a Leica-421565 ﬂuorescence microscope (Germany).

### Transmission electron microscope investigation

For transmission electron microscopy, the renal tissues were briefly rinsed with phosphate buffered saline (PBS) and were then fixed using 2.5% glutaraldehyde at 4 °C. Then, 1% osmium tetroxide was used for secondary fixation for 1 hour at 4 °C. After washing with PBS, the samples were treated with 1% uranyl acetate at 4 °C for 1 hour; then, they were dehydrated in a series of graded ethanol solutions. The samples were then embedded in epoxy resin. Ultrathin sections of the tissue samples were double stained with uranyl acetate and lead citrate. The sections were evaluated under a JEM1200EX electron microscope (HITACHI, JAPAN) at 80 keV.

### Statistical analysis

The WB analyses and the cell viability assessment were repeated at least 3 times independently, and the individual data were subjected to a densitometric analysis. The data are expressed as the means ± SD. The statistical analysis was performed using the non-parametric Mann-Whitney U test to compare the data in the different groups. A *P* value < 0.05 indicated a statistically significant difference.
