# Correction for: Hyperoside attenuates renal aging and injury induced by D-galactose via inhibiting AMPK-ULK1 signaling-mediated autophagy

**DOI:** 10.18632/aging.205888

**Published:** 2024-05-15

**Authors:** Buhui Liu, Yue Tu, Weiming He, Yinglu Liu, Wei Wu, Qijun Fang, Haitao Tang, Renmao Tang, Ziyue Wan, Wei Sun, Yigang Wan

**Affiliations:** 1 Department of Traditional Chinese Medicine, Nanjing Drum Tower Hospital Clinical College of Traditional Chinese and Western Medicine, Nanjing University of Chinese Medicine, Nanjing 210008, China; 2 Department of Nephrology, The Affiliated Hospital of Nanjing University of Chinese Medicine, Nanjing 210029, China; 3 Department of TCM Health Preservation, Second Clinic Medical School, Nanjing University of Chinese Medicine, Nanjing 210023, China; 4 Department of Traditional Chinese Medicine, Nanjing Drum Tower Hospital, The Affiliated Hospital of Nanjing University Medical School, Nanjing 210008, China; 5 Institute of Huangkui, Suzhong Pharmaceutical Group Co., Ltd., Taizhou 225500, China; 6 Department of Social Work, Meiji Gakuin University, Tokyo 108-8636, Japan

**Keywords:** hyperoside, renal aging, autophagy, AMPK-ULK1 signaling pathway, mTOR signaling pathway, vitamin E

**This article has been corrected:** The authors found that the Western blot image for GAPDH in **Figure 4A** and the transmission electron micrograph for the D-gal plus vitamin E group in **Figure 6C** were incorrect due to misuse of data from a different experiment. The authors have provided uncropped images of the original blots and electron micrographs from three sets of experiments and replaced the incorrect images with images from the original experiments. These corrections have no impact on the experimental outcomes or conclusions.

New **Figure 4** and **Figure 6** are presented below.

**Figure 4:**
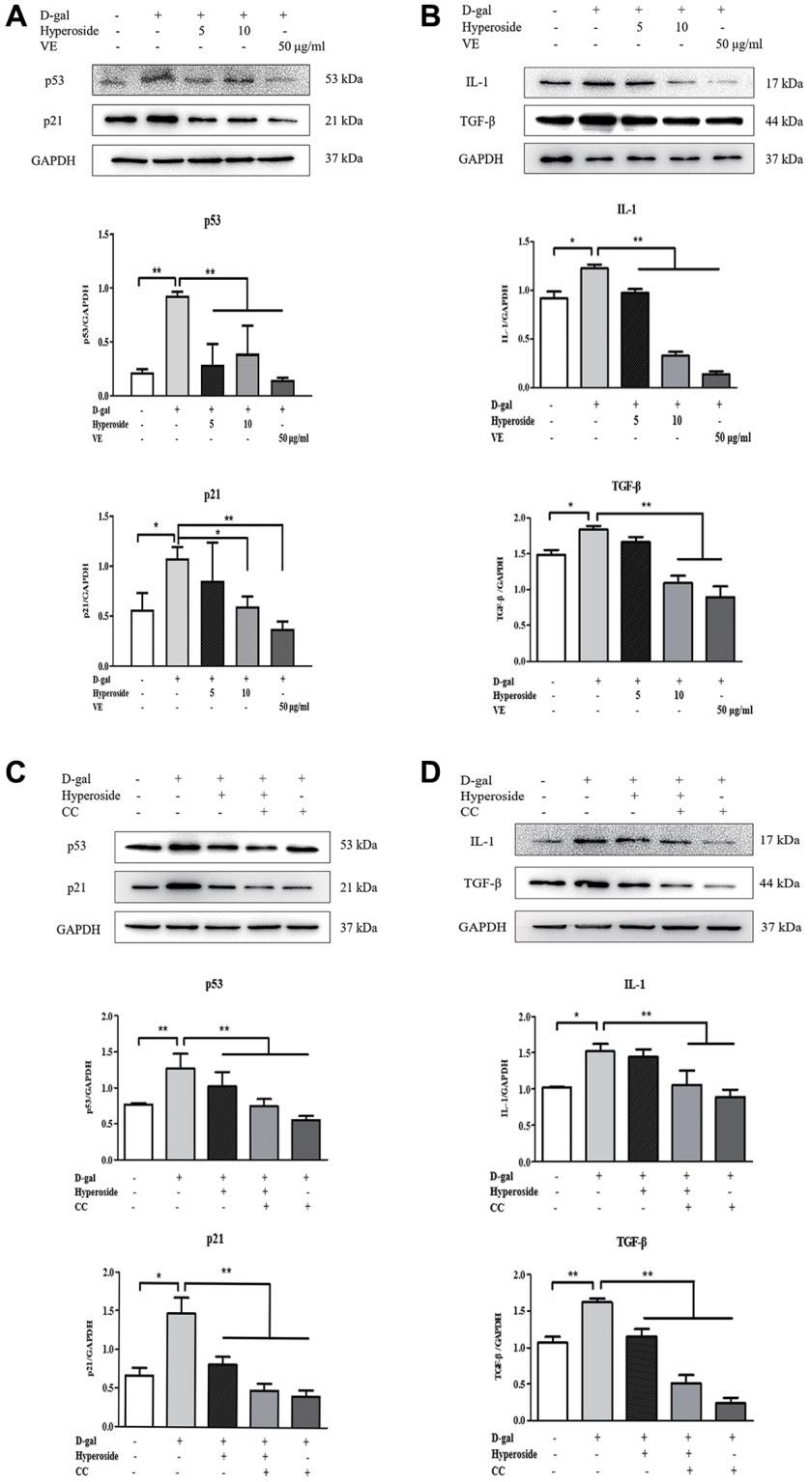
**The actions of hyperoside, vitamin E and compound C on renal cellular aging and injury *in vitro*.** (**A**, **B**) The NRK-52E cells were exposed to D-gal at 100 mM, with the treatment of hyperoside at 0, 5, and 10 μg/ml and VE at 50 μg/ml for 24 hours, and subjected to a WB analysis for p53, p21, IL-1 and TGF-β, respectively. (**C**, **D**) The NRK-52E cells were exposed to D-gal, with the treatment of hyperoside and CC for 24 hours, and subjected to a WB analysis for p53, p21, IL-1 and TGF-β, respectively. The data are expressed as the mean ± SD, (*n* = 3), ^*^P < 0.05, ^**^P < 0.01. Abbreviations: D-gal: D-galactose; VE: vitamin E; WB: Western blot; IL-1: interleukin-1; TGF-β: transforming growth factor-β; CC: compound C.

**Figure 6:**
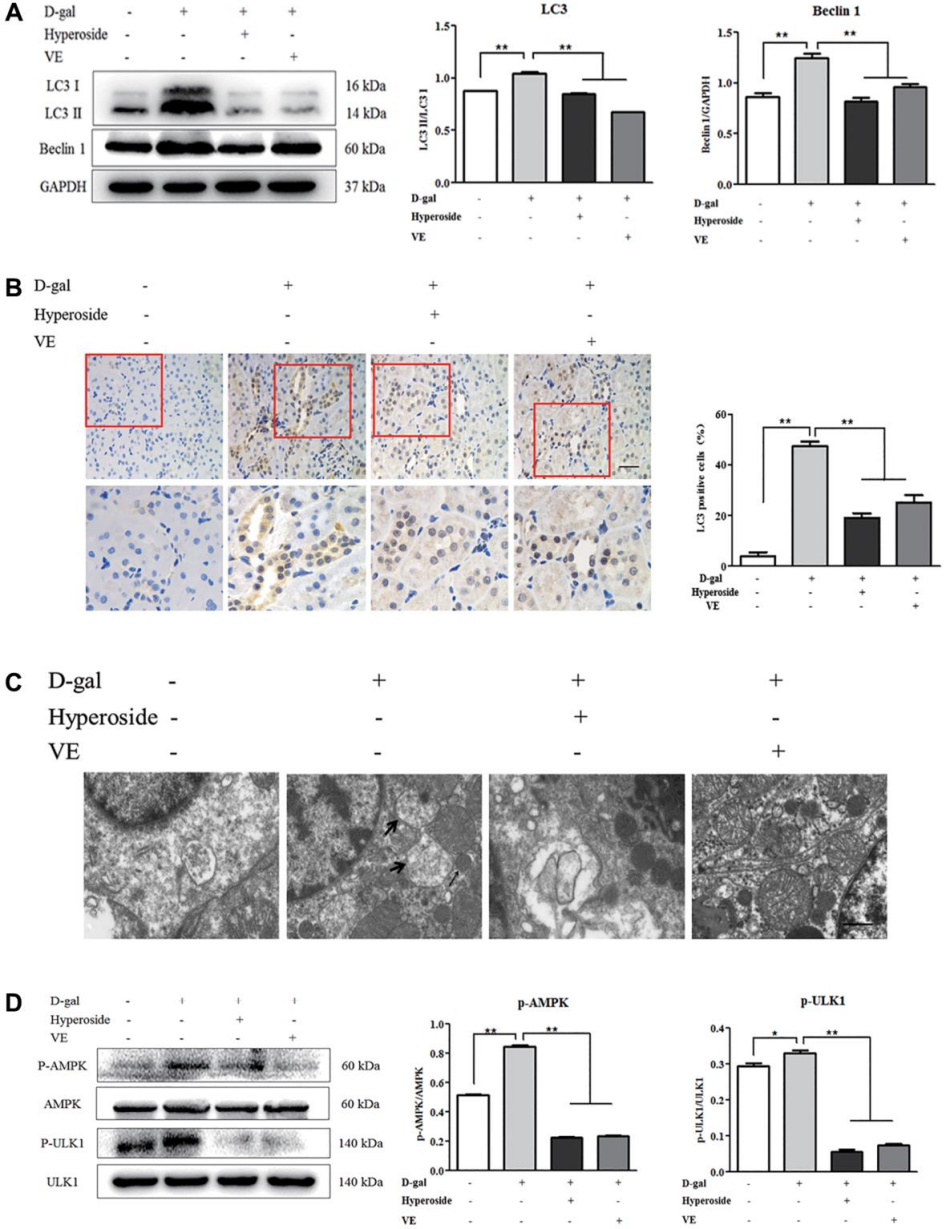
**The effects of hyperoside and vitamin E on autophagic activity and the AMPK-ULK1 signaling pathway *in vivo*.** (**A**) A WB analysis of LC3 I/II and Beclin1 in the kidneys from the rats in the control, the 8 week-D-gal, the D-gal + Hyperoside and the D-gal + VE groups. (**B**) Immunohistochemical staining of LC3 and the percentage of the positively stained areas of LC3 in the control, the 8 week-D-gal, and the D-gal + Hyperoside groups. Scale bar = 20 μm. (**C**) The morphological changes in the renal tubular cells of the rats in the control, the 8 weeks-D-gal, the D-gal + Hyperoside and the D-gal + VE groups by transmission electron microscopy. The black arrows show the autophagosomes with the characteristic morphology of a double membrane. (**D**) A WB analysis of p-AMPK, AMPK, p-ULK1 and ULK1 in the kidneys of the rats in the control, the 8 weeks-D-gal, the D-gal + Hyperoside and the D-gal + VE groups. The data are expressed as the mean ± SD, (*n* = 3), ^*^P < 0.05, ^**^P < 0.01. Abbreviations: WB: Western blot; D-gal: D-galactose; VE: vitamin E; p-AMPK: phosphorylated AMPK; p-ULK1: phosphorylated ULK1.

